# Diagnostic accuracy of the ‘Dysphagia Screening Tool for Geriatric Patients’ (DSTG) compared to Flexible Endoscopic Evaluation of Swallowing (FEES) for assessing dysphagia in hospitalized geriatric patients – a diagnostic study

**DOI:** 10.1186/s12877-023-04516-7

**Published:** 2023-12-14

**Authors:** Ulrich Thiem, Martin Jäger, Holger Stege, Rainer Wirth

**Affiliations:** 1 Albertinen-Haus Hamburg, Sellhopsweg 18-22, 22459 Hamburg, Germany; 2 Geriatrics and Gerontology, Medical Center Hamburg-Eppendorf (UKE), Hamburg, Germany; 3 Hüttenhospital gemeinnützige GmbH, Am Marksbach 28, 44269 Dortmund, Germany; 4 Department of Geriatrics, ukrb University Clinic Ruppin-Brandenburg, Fehrbelliner Straße 38, 16816 Neuruppin, Germany; 5 Department of Geriatrics, Marien Hospital Herne, University Hospital of the Ruhr-University Bochum, Hölkeskampring 40, 44625 Herne, Germany; 6 Chair of Geriatrics, Ruhr-University Bochum, Bochum, Germany

**Keywords:** Geriatrics, Dysphagia, Swallowing disorder, Deglutition, Deglutition disorder, Geriatric assessment, Diagnostic test, Flexible endoscopic evaluation of swallowing

## Abstract

**Background:**

Oropharyngeal dysphagia is highly prevalent among hospitalized geriatric patients. The screening instruments used to date have been evaluated primarily in stroke patients. This diagnostic study aimed to validate a new screening instrument for oropharyngeal dysphagia, the ‘Dysphagia Screening Tool for Geriatric Patients’ (DSTG), as compared to one of the gold standards, flexible endoscopic evaluation of swallowing (FEES).

**Materials and methods:**

Geriatric inpatients admitted to five geriatric hospitals in Germany were consecutively evaluated using both DSTG and FEES in random order and by different evaluators blinded to the results of the other evaluation. In the FEES examination, a score of more than 3 on Rosenbek’s Penetration Aspiration Scale was considered evidence of clinically relevant oropharyngeal dysphagia. Sensitivity, specificity and further measures of test performance were calculated for DSTG.

**Results:**

The 53 volunteers recruited were on average 85 years of age, 56.6% were women. Twenty patients (37.7%) were diagnosed with dysphagia using FEES. Of these, 12 were screened as positive on DSTG. Of the 33 FEES negative patients, 4 tested positive on DSTG. The following test parameters were calculated for DSTG: sensitivity: 0.60, 95% confidence interval [0.39 ; 0.78], specificity 0.88 [0.73 ; 0.95], positive predictive value 0.75 [0.51 ; 0.90], negative predictive value 0.78 [0.63 ; 0.89], positive likelihood ratio 4.95 [1.85 ; 13.27], negative likelihood ratio 0.46 [0.26 ; 0.79]. In a receiver-operator characteristic (ROC) curve analysis, the area under the curve (AUC) was 0.77 [0.62 ; 0.91]. No adverse events occurred.

**Conclusion:**

The DSTG appears to be a valid instrument for screening of oropharyngeal dysphagia in geriatric inpatients.

## Introduction

Swallowing is a complex function that transports saliva and food from the oral cavity to the stomach, including airway protection. Swallowing involves a wide range of structures and operations, including oropharyngeal sensory function, an extensive neuronal swallowing network and highly coordinated action of the small muscles of the throat and neck. This complex function makes the system vulnerable, however, often causing oropharyngeal dysphagia, especially among geriatric patients and patients with neurological impairment [1].

Oropharyngeal dysphagia is defined as any swallowing dysfunction [2, 3]. While the definition includes all possible clinical presentations and findings, it does not require the patient’s conscious awareness of the condition and thus also includes silent aspiration. Silent aspiration without cough reflex is a frequent finding which occurs in about one third of patients with aspiration [4, 5]. Aspiration in general puts affected patients at increased risk for pneumonia [6–9]. Oropharyngeal dysphagia is associated with complications which can be very severe [10] and frequently lead to impaired quality of life and increased morbidity and mortality [11–13]. Due to the multifactorial etiology and substantial clinical consequences, oropharyngeal dysphagia was declared a geriatric syndrome in 2016 [14, 15].

The prevalence of oropharyngeal dysphagia among seniors living independently is between 20% and 30% [15–17]. Around 40–50% of older people living in care facilities are affected [17]. In geriatric hospital patients in Germany in whom stroke, Parkinson’s disease or dementia were the primary condition, the prevalence of oropharyngeal dysphagia is around 40% [18]. Based on instrumental examination, the prevalence of dysphagia was 48% among patients 60 years and older consecutively admitted to an acute care hospital in the United States [19]. The prevalence of dysphagia in consecutively admitted patients with pneumonia was more than 50% after clinical diagnosis [20].

A multi-level approach entailing screening, clinical assessment and instrumental examination methods has become established for diagnosing dysphagia and risk stratification; the components build on each other sequentially. Screening is used for risk stratification for dysphagia, and clinical assessment is used to identify the presence and severity of dysphagia. Instrumental examination captures objective data about the presence, pattern, severity and pathogenesis of dysphagia to provide a basis for targeted therapeutic measures. In the Scottish Intercollegiate Guidelines Network (SIGN) guideline for the identification and management of dysphagia in patients with stroke (2010), screening and clinical assessment are assigned recommendation grade B [21]. In the 2013 guideline of the German Society for Nutritional Medicine (DGEM), Clinical Nutrition in Neurology [22], the screening method of the SIGN Water Swallow Test [21] and the Gugging Swallowing Screen (GUSS) [23] involving multiple consistencies are recommended. The guidelines for neurogenic dysphagia published by the German Society of Neurology (DGN) [24] state that the above-mentioned screening tests are well validated for patients for whom stroke is the primary disorder, but not for other age-associated disorders such as Parkinson’s disease, dementia disorders, malnutrition, frailty, sarcopenia or geriatric multimorbidity. The volume-viscosity swallow test (VVST) is an additional screening tool that has been validated for older patients with pre-existing dysphagia [25]. As a test involving multiple consistencies, the VVST is relatively time-consuming for routine clinical screening.

As a feasible and validated screening tool for geriatric patients is lacking, routine dysphagia screening is not yet standard in geriatric hospital care. The goal of the study reported here was to evaluate the new ‘Dysphagia Screening Tool for Geriatric Patients’ (DSTG) [26] and validate it against the flexible endoscopic evaluation of swallowing (FEES) as a gold standard.

## Materials and methods

### Inclusion and exclusion criteria

This prospective diagnostic study included patients aged 70 years and older who had been newly admitted for inpatient treatment at a geriatric acute care hospital. Further inclusion criteria were hospitalization for at least 72 h and written consent for participation in the study. Patients with contraindications for dysphagia screening or FEES, i.e. established dysphagia diagnosis or complications in prior endoscopy and/or FEES examination, in palliative care situation, with unwillingness or inability to participate in the project-related data acquisition, or failing to provide written consent were excluded from the study.

### Data acquisition

When including patients in the study, the following variables were recorded: age, sex, date of admission for inpatient treatment, primary diagnosis, comorbidities as defined by the Charlson Comorbidity Index as modified by Quan [27], the Barthel Index (German version in accordance with the Hamburg Classification Manual) [28], dysphagia and, if present, purported causes of dysphagia, and quality of life (visual analogue scale from EQ-5D) [29]. The results of DSTG screening and FEES were also recorded during the course of the study.

### Study organization and implementation

Patient study candidates were to be recruited and included at the participating hospitals consecutively and independently of the reason for admission. After the inclusion and exclusion criteria were reviewed, patients provided written informed consent, and data collection of patient characteristics was performed. Subsequently, both DSTG screening and FEES were scheduled and performed in a timely manner. The order of the tests, either DSTG screening first or FEES first, was randomized using block randomization with variable block size (block size between 4 and 8) and stratified according to the recruiting hospital. Randomization was performed centrally at the study office. After the participants were recruited, registered and pseudonymized, the office reviewed the inclusion and exclusion criteria, then included and randomized the patients in the study, and finally faxed the results of the randomization to the respective hospital. The DSTG screening and FEES were performed on the same day or within the first two days after randomization by different individuals blinded to the results of the other test. No dysphagia-related interventions were performed between the two tests.

### Performance of DSTG and FEES

Standardized DSTG screening training material was prepared by the Working Group on Dysphagia and was used to standardize performance of DSTG. The training content was presented by the head of the working group (MJ) at a meeting of all of the heads of participating centers. The heads of the centers trained study staff at their respective centers. Only geriatric hospitals with examiners experienced with dysphagia, meaning qualified medical personnel or speech therapists, were permitted to participate in the study. For standardization of FEES, each hospital was presumed to have an existing plausible test standard and examiners with several years of experience.

The FEES examination following the Langmore standard protocol [30, 31] contained different consistencies, e.g. thin liquids (water), thick liquids of nectar like consistency, puree, solid food, all dyed with food coloring. The FEES findings were evaluated using Rosenbek’s Penetration-Aspiration Scale (PAS, German version) [32, 33] in which a PAS score of greater than 3 was rated as a criterion for endoscopic detection of clinically relevant dysphagia. It was anticipated that a higher cut-off, for example of 6, the threshold for actual aspiration, would probably lead to better test performance in terms of sensitivity etc. However, a meaningful clinical consequence from screening would be taking precautions not only in patients with actual aspiration, but also in patients with considerable risk for aspiration or in suspected cases. Therefore, a score of greater than 3 was chosen.

The Working Group on Dysphagia of the German Society of Geriatrics (Deutsche Gesellschaft für Geriatrie, DGG) has designed and approved a screening method for geriatric patients entitled the ‘Dysphagia Screening Tool for Geriatric Patients’ (DSTG) [26]. It includes three criteria for evaluation: 1. ‘Overall condition’ (sufficient alertness and posture control while sitting), 2. ‘Oral inspection’ (observation of swallowing saliva, adequate tongue mobility and adequate ability to cough on request), 3. ’Water swallow test’ (clearing the throat, coughing or change of voice up to one minute after ingesting a small amount of water from a teaspoon [5 ml], then from a glass [30 ml]). If any one of the criteria for ’Overall condition‘ and ’Oral inspection‘ and for clearing the throat, coughing or change of voice in the Water Swallow Test were not met, the screening was considered positive and the participant was suspected to have dysphagia.

### Sample size estimation and statistics

For the sample size estimation, we assumed 75% sensitivity and specificity for the DSTG and a lower margin of the confidence interval of 65% for both. We expected a dysphagia prevalence of 40% for a mixed geriatric inpatient population. Using an approach of sensitivity-based sample size calculation [34] with an alpha level of 5%, a total sample size of 180 participants was calculated, 72 participants with and 108 participants without dysphagia.

The characteristics of the participants are described qualitatively, categorical variables are expressed as absolute and relative frequencies, and continuous variables are expressed as mean and standard deviation, median, and minimum/maximum. Sensitivity and specificity, positive and negative predictive values, as well as positive and negative likelihood ratios, each with the associated 95% confidence interval, were calculated to assess the quality of DSTG as a diagnostic test. Receiver-operator characteristic (ROC) curve analysis was also performed. Confidence intervals were calculated using the software Confidence Interval Analysis (CIA, Trevor Bryant, University of Southampton, 2011). All other analyses were performed with SPSS for Windows (version 22, 2014, IBM Inc., USA).

### Ethics

Epidemiological and clinical studies are governed by the principles of research on human subjects. The study protocol was thus presented for review to the Ethics Committee of the Medical Faculty of the Ruhr-Universität Bochum, Germany. It received a positive vote (Vote no. 16-5661, dated June 20, 2016). The study was performed in accordance with the Declaration of Helsinki (2013 version) and the recommendations for ‘Good Epidemiologic Practices of the German Working Group on Epidemiological Methods of the German Epidemiological Foundation’ (DAE) (internet: http://dgepi.de/berichte-und-publikationen/leitlinien-und-empfehlungen.html) and in compliance with the relevant legal provisions, including data protection provisions. The study protocol and additional documentation are available from the authors.

## Results

Five geriatric hospitals in Germany recruited volunteers for the study starting on October 1, 2016: GFO Kliniken Rhein-Berg, Bergisch-Gladbach, Ruppiner Kliniken, Neuruppin, GFO Kliniken Troisdorf, Marienhospital Brühl, Marienhospital Herne. The recruitment markedly slowed after six months. Feedback on recruitment results and the proceeding of the study was given to recruiting sites, responsible physicians were contacted and motivated to recruit further, and efforts were made to involve further clinics potentially interested in study participation. However, due to limited success of all efforts, the study was discontinued on September 1, 2017, because of low recruitment.

A total of 64 participants were informed and included in the study after providing their written consent. Of these participants, 11 had to be excluded for several reasons: 8 participants refused to undergo FEES testing, 2 participants were retroactively found to have exclusion criteria, and 1 participant had no study documentation available for evaluation. For the remaining 53 patients, data were complete.

Table 1 shows the characteristics of the 53 evaluated patients. The average age was over 85 years, and 56.6% (30 of 53) were female. The following primary diagnoses were found: acute or subacute stroke (13 patients, 24.5%), fractures (hip, humeral or vertebral fracture, 10 patients, 18.9%), respiratory tract infection (9 patients, 17.0%), abnormalities of gait and mobility (5 patients, 9.4%), Parkinson’s disease (3 patients, 5.7%), others (13, 24.5%). Among secondary diagnoses, cerebrovascular disease (28 patients, 52.8%), diabetes (19 patients, 35.8%), cognitive impairment / dementia (18 patients, 34.0%), and chronic heart failure (13 patients, 24.5%) were the most common. When present, dementia was mild to moderate at most. In general, the population characteristics match those of a typical geriatric sample of elderly inpatients with advanced comorbidity and significant functional impairment.

**Table 1:** Characteristics of the study population (n = 53)

Variable	Mean	Standard deviation	Minimum	Maximum	Median
Age (years)	85.6	± 6.0	74	98	86.0
Barthel Index (points)^a^	39.4	± 19.4	5	80	35.0
Number of comorbidities	2.5	± 1.3	0	5	2.0
Charlson Comorbidity Index (points) ^b^	2.6	± 1.9	0	8	2.0
Quality of life (VAS) ^c^	50.1	± 19.4	10	90	50

^a^ Barthel Index (Hamburg Classification Manual) [20]: number of points from 0 to 100, higher values show greater independence in performing basic activities of daily living; ^b^ Charlson Comorbidity Index as modified by Quan [27]: weighted index of comorbidity, higher values represent more severe comorbidity; ^c^ quality of life as measured by the visual analog scale (VAS) [21]: values from 0 to 100 possible, higher values indicate better quality of life

The prevalence of dysphagia diagnosed by FEES was 37.7% (20 out of 53). Both tests – DSTG screening and FEES testing – gave unequivocal findings. Application of the DSTG took about three to five minutes for instructed personnel. No side effects or complications were noted for either test. In about a quarter of patients, both tests were performed on the same working day, i.e. within eight hours. For the remaining patients, the second test was done on the following day, within 24 to 32 h (maximum).

**Fig. 1:**
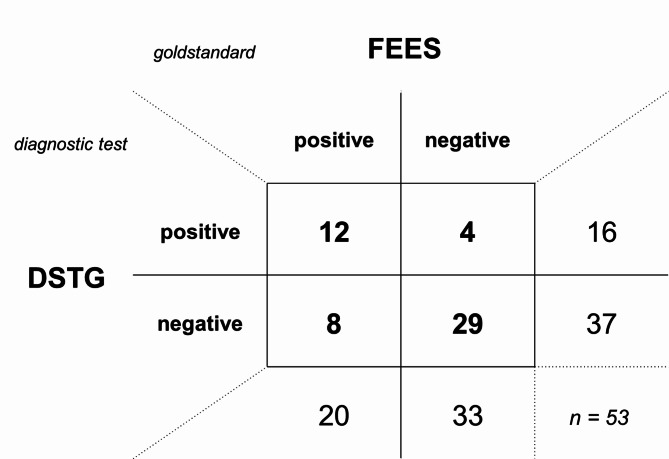
Contingency table comparing DSTG (diagnostic test) to FEES (gold standard) Legend: *DSTG* Dysphagia Screening Tool for Geriatric Patients, *FEES* flexible endoscopic evaluation of swallowing

**Fig. 2:**
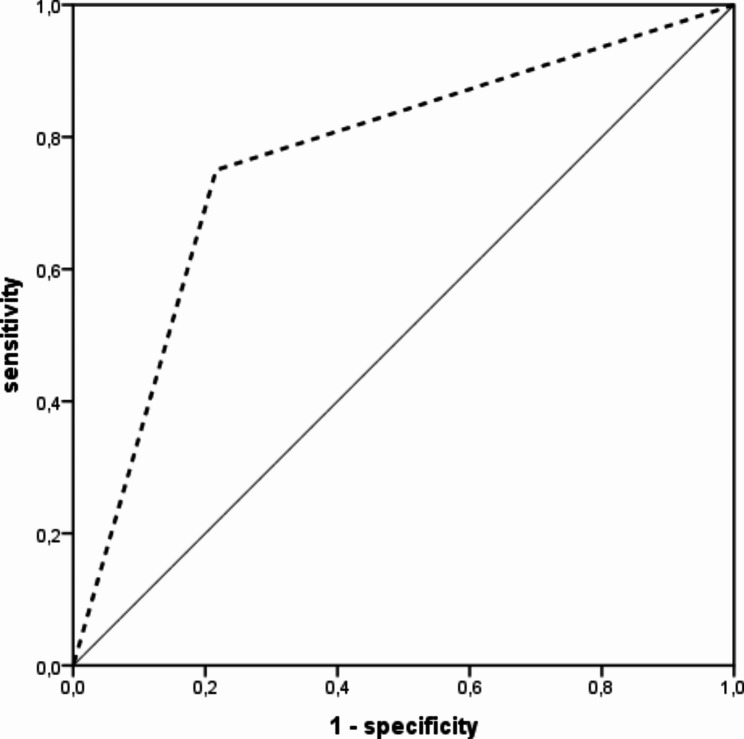
Receiver operator characteristic (ROC) curve analysis of DSTG (diagnostic test) compared to FEES (gold standard) Legend: *DSTG* Dysphagia Screening Tool for Geriatric Patients, *FEES* flexible endoscopic evaluation of swallowing, AUC (area under the ROC curve): 0.77 95% confidence interval [ 0.62 ; 0.91 ], (p = 0.002)

DSTG identified 12 of the 20 total patients with dysphagia as having dysphagia (Fig. 1). Of the 33 patients testing negative for dysphagia on FEES, 4 showed abnormal results in DSTG. Sensitivity was 60% and specificity was 88% (Table 2). With a positive likelihood ratio, the probability of dysphagia was approximately five-fold higher in DSTG-positive patients. The probability of dysphagia diminished by around 50% in case of a negative DSTG (negative likelihood ratio). In the receiver-operator characteristic (ROC) curve analysis, the area under the curve (AUC) was 77% (95% confidence interval: 62%; 91%, see Fig. 2). Investigating the three components of the DSTG, overall condition, oral inspection and Water Swallow Test, separately, the worst test performance was found for overall condition, and the best for the Water Swallow Test. However, none of the three components performed as well as the overall test (data not shown). Table 3 presents the frequency of DSTG abnormalities by DSTG areas and criteria. Based on this, abnormalities were most common in the Water Swallow Test, followed by the area ’Overall health‘ (alertness and seated position).

**Table 2:** Characteristics of the Dysphagia Screening Tool for Geriatric Patients (DSTG) as a diagnostic test

Statistical measure	Estimate	95% confidence interval
Sensitivity	0.60	[0.39 ; 0.78]
Specificity	0.88	[0.73 ; 0.95]
Positive predictive value (PPV)	0.75	[0.51 ; 0.90]
Negative predictive value (NPV)	0.78	[0.63 ; 0.89]
Positive likelihood ratio (LR+)	4.95	[1.85 ; 13.27]
Negative likelihood ratio (LR-)	0.46	[0.26 ; 0.79]

**Table 3:** Abnormalities in DSTG based on individual DSTG criteria

Criterion ^a^	Frequency	Proportion ^b^
Alertness	4	7.5%
Postural position, sitting	2	3.8%
Swallowing saliva	0	0%
Tongue mobility	1	1.9%
Coughing upon request	6	11.3%
Coughing after water swallow	13	24.5%
Voice change	8	15.1%
Total number of cases with abnormalities	16	30.2%

^a^ More than one per case possible; ^b^ based on n = 53 cases

## Discussion

The results of this initial study on a new tool for dysphagia screening are promising and indicate that DSTG may be a valid clinical tool for detecting oropharyngeal dysphagia in hospitalized geriatric patients. The DSTG is comparable in sensitivity and specificity to other tools described in the literature. The Water Swallow Test as described by Daniels, which is widely used in geriatric hospitals in Germany, has a sensitivity of 92% and a specificity of 67% [35]. For other tools, sensitivities between 42% and 85% and specificities between 45% and 92% are known [36–39]. For VVST and GUSS, better sensitivities (84–100%) are reported compared to DSTG, with poorer specificities, however (50–67%) [23, 25, 40]. For the SIGN Water Swallow Test, the sensitivity varies between 76% and 92%, and the specificity between and 22% and 67%, respectively [21]. Rofes et al. reported very good test properties for the VVST (sensitivity 94%, specificity 88%) [40].

A key difference between the aforementioned studies and the evaluation reported here is the patient population in which they were carried out. Our study included only geriatric hospitals that included older inpatients independent of the reason for admission or primary diagnosis. The inclusion of patients was performed consecutively and thus nonselective by principle. As described in the results section, the characteristics of the participants included here match those of typical geriatric inpatients. In contrast, in many comparable studies the patient groups tended to be selective. The study carried out by Daniels [35] included only patients in the acute phase of stroke treatment. Stroke populations were also the target group in a number of other studies [21, 23, 36–39]. Older patients with dysphagia were also included in the evaluation of the VVST [25, 40]; however, stroke and neurodegenerative disorders were the predominant primary diagnoses in these studies, so here, too, patients were most likely to have a neurological primary disorder. This focus leads the authors of German guidelines for neurogenic dysphagia to conclude that statements on the value of dysphagia screening tools for patients other than stroke patients are therefore nearly impossible [24].

Deviations in the results of our study from results reported in the literature can be further explained by additional differences between studies. For example, several comparable studies were performed in rehabilitative medicine [36, 38, 39] or in mixed cohorts [21, 38] rather than in acute care medicine. Similar to our study, Trapl et al. selected FEES as the gold standard [23]. In nearly all other studies, clinical screening was compared to video fluoroscopy as the gold standard. The patient cohort used to validate the VVST included older patients with known swallowing impairment in their history, who therefore had a higher a priori probability of the presence of dysphagia [25, 40].

An important difference between a Water Swallow Test, VVST, and DSTG is the way the swallow test was performed. The repeated performance of swallow tests with different volumes [35] and different consistencies (VVST) [25, 40] enables better and more accurate clinical assessment of existing oropharyngeal dysphagia. Against this background, better test properties, as reported by Rofes [40] for example, compared to the DSTG would not be surprising. However, the working group that developed and approved DSTG deliberately decided not to use different volumes and consistencies in favor of saving time. Tests must be feasible, practical and reliable to enjoy widespread routine clinical use. One way to improve the DSTG test properties reported here would be to add more clinically significant and predictive clinical signs of dysphagia. However, the working group is not aware of any additional significant clinical signs that could be integrated into the test quickly and without major extra effort.

The value of DSTG for clinical use may best be illustrated by likelihood ratios. With a positive likelihood ratio, oropharyngeal dysphagia as confirmed on FEES is around five times more likely, while the likelihood ratio is reduced to around one-half for a negative test result. In general, diagnostic tests are sufficiently meaningful if the likelihood of the presence of a state to be diagnosed is at least doubled or (negative test results) halved [41]. This is the case for DSTG.

The advantages of the study reported here involve the use of screening for typical geriatric patients, the multicentric approach of the study with five recruiting centers, and the randomization of the order of the dysphagia screening test (diagnostic test) and FEES (gold standard), which makes systematic bias in favor of one of the two methods unlikely. However, the study also has several limitations that must be taken into account when interpreting the results. First, the slow patient recruitment meant that the originally targeted sample size was not achieved. This is not related to the DSTG itself, which did not result in any considerable complication. Acceptance by health care personal was reported to be good. However, the precision of the estimator of the identified test properties, as can be seen with relatively broad confidence intervals, is reduced. Larger studies that determine the estimators of the test quality with more precision are therefore needed. Second, it was not feasible to include all of the newly admitted and eligible patients in the participating hospitals. This is necessary to ensure that the DSTG is applied to a truly geriatric inpatient population. We are still confident, however, that the study population reported here is much more similar to typical geriatric inpatients than the populations of other comparable dysphagia studies. Third, the test performance could be improved by adding swallow tests with different volumes and/or different consistencies to the DSTG, or to additionally measure the maximum swallowing volume. However, as mentioned above, we elected not to do this when developing and approving a feasible screening test. Finally, people with severe cognitive impairment and/or advanced dementia were not present in the study population, so the findings of the study may not be applicable to this group of people.

The data indicate that the Dysphagia Screening Tool for Geriatric Patients (DSTG) is possibly a sufficient tool for the screening of oropharyngeal dysphagia in hospitalized geriatric patients. Additional studies are needed to investigate the tool further, and to evaluate whether using the tool will improve geriatric inpatient care and safety.

## Data Availability

Data from this study are not publicly available due to legal issues, especially data protection regulations. However, data can be made available by the authors upon reasonable request.

## Abbreviations

AUC: area under the curve
CI: confidence interval
DGG: German Geriatrics Society (Deutsche Gesellschaft für Geriatrie)
DSTG: Dysphagia Screening Tool for Geriatric Patients
FEES: flexible endoscopic evaluation of swallowing
GUSS: Gugging Swallowing Screen
ml: milliliter
PAS: (Rosenbek’s) Penetration-Aspiration Scale
ROC: receiver-operator characteristic
SIGN: Intercollegiate Guidelines Network
VVST: volume-viscosity swallow test
